# Acute Necrotizing Ulcerative Gingivitis: A Case Report

**DOI:** 10.7759/cureus.63023

**Published:** 2024-06-24

**Authors:** Monitha Gollapudi, Swapnil Mohod, Neha Pankey, Pranjali Gatlewar

**Affiliations:** 1 Periodontology, Sharad Pawar Dental College and Hospital, Datta Meghe Institute of Higher Education and Research, Wardha, IND; 2 Oral Medicine and Radiology, Sharad Pawar Dental College and Hospital, Datta Meghe Institute of Higher Education and Research, Wardha, IND; 3 Pediatric and Preventive Dentistry, Sharad Pawar Dental College and Hospital, Datta Meghe Institute of Higher Education and Research, Wardha, IND; 4 Dentistry, Sharad Pawar Dental College and Hospital, Datta Meghe Institute of Higher Education and Research, Wardha, IND

**Keywords:** nutritional status, alveolar bone loss, impaired host defenses, virulent bacteria, acute necrotizing ulcerative gingivitis

## Abstract

Compared to other conditions found in the necrotizing periodontal diseases group, acute necrotizing ulcerative gingivitis (ANUG) is a definite and specific disease. This illness has a long history that originates from the time of Hippocrates and is also referred to by several synonyms. ANUG occurs less commonly than other oral disorders, even though it is typically not rare. It starts suddenly, advances quickly, and finally results in significant loss of alveolar bone and soft tissue. Viral microorganisms and weakened host defenses have been linked to the etiology and pathophysiology of ANUG. In situations where there is psychological and physiological stress, the incidence of ANUG rises. In developed nations, the incidence of ANUG has declined and, in some cases, gone extinct due to the development of antibiotics and improved nutritional status. However, due to the persistently low nutritional status, the illness continues to be a frequently diagnosed clinical lesion in developing nations. This case report presents the case of a 24-year-old ANUG patient and the sequential treatment of this patient.

## Introduction

Military historians have been recording painful, bleeding gingival tissues with fetor oris and associated necrosis for millennia. This disease entity was first documented during the time of Hippocrates, and later, in the 1890s, Plaut and Vincent scientifically investigated this oral disease. However, this infectious and contagious oral disease was an outbreak among combat troops and returning military personnel during World War I and II. It was referred to as the trench mouth [1]. The disease has a lower rate in the common population. A considerable amount of literature on the general population is mainly focused on patients with HIV and severely malnourished children. Only a few cases have been reported in oncology patients, whereas no cases are reported in patients with bone tumors or sarcoma [2].

The American Academy of Periodontics defined acute necrotizing ulcerative gingivitis (ANUG) as a kind of necrotizing periodontal disease in 1999. The symptoms of ANUG include acute periodontal tissue damage and progressive ulceration that usually begins at the tip of the interdental papilla and spreads along the gingival margin. The diagnosis is mainly based on clinical findings, namely, the presence of interdental papilla necrosis pain, pseudomembranous formation accompanied by a fetid odor, and acute necrosis leading to an inverted crater-like interdental papilla. Symptoms such as lymphadenopathy, fever, and malaise are frequently seen [3].

Although the exact etiology of ANUG remains unknown, it is thought to be caused by a polymicrobial infection in which commensals of the oral cavity are the implicated organisms. However, the organisms turn pathogenic when the gingival area's local resistance diminishes. *Fusobacterium necrophorum*, *Bacteroides melaninogenicus *species intermedius (also known as *Prevotella intermedia*), *Porphyromonas gingivalis*, *Fusobacterium nucleatum*, *Selenomonas*, and *Treponema* species are among the anaerobic organisms that have been identified as significant contributors to ANUG. Numerous damaging and hazardous metabolites, including endotoxins, hydrogen sulfide, indole ammonia, fatty acids, collagenase, fibrinolysin, and protease, are produced by these *Bacteroides* species. These metabolites can break down immunoglobins and additional substances that prevent neutrophil and chemotaxis [4]. In an undernourished and immunocompromised population such as children and HIV patients, failure to provide the necessary treatment regimen for ANUG can result in the progression of the disease to necrotizing periodontitis. Therefore, immediate and appropriate management in the initial days is crucial [5].

Over the past few years, there has been a significant need for additional research on ANUG, mainly from the perspective of its contribution to the occurrence of cancrum oris, which is described as a neglected third-world disease [3]. This is a case presentation of a 24-year-old male presenting with ANUG, having a known history of betel squid chewing (a mixture of tobacco, crushed areca nuts, spices, and other ingredients, including lime) and stress.

## Case presentation

**Figure 1:**
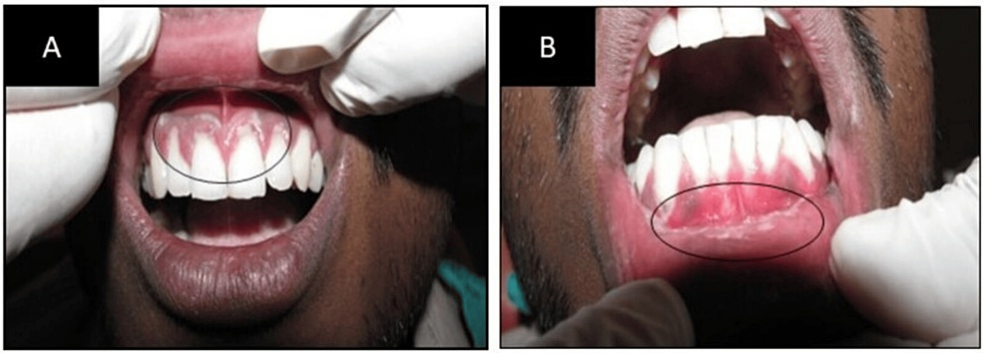
(A) Pseudomembrane is observed in the upper anterior region of the jaw; (B) pseudomembrane is seen in the lower anterior region of the jaw

A 24-year-old male came to the Department of Oral Medicine and Radiology at Sharad Pawar Dental College and Hospital, Datta Meghe Institute of Higher Education and Research (DMIHER), with a chief complaint of burning sensation, pain, and bleeding from gums in the upper and lower anterior teeth regions of the jaw. The patient also gave a history of headaches, malaise, and difficulty brushing his teeth. There was no difficulty in mastication or a change in phonetics. The patient was a betel quid chewer, consuming it seven to eight times daily since the age of 22. Additionally, he reported experiencing sleep disturbances during the past three weeks due to stress. On extra examination, the right submandibular lymph nodes are palpable, approximately 2x1 cm, soft, mobile, and tender. On intraoral examination, generalized inflammation and erythema are seen in the jaw's upper and lower anterior regions. Pseudomembrane, which is scrapable, is also seen on the gingiva (Figure 1). A complete blood count (CBC) with differential analysis was conducted to look for potential connections to systemic illnesses or infections, such as HIV or other microbial infections. The test findings showed a slightly lower lymphocyte count (15.4%) and a higher neutrophil percentage (81%), indicating an acute infection state.

**Figure 2:**
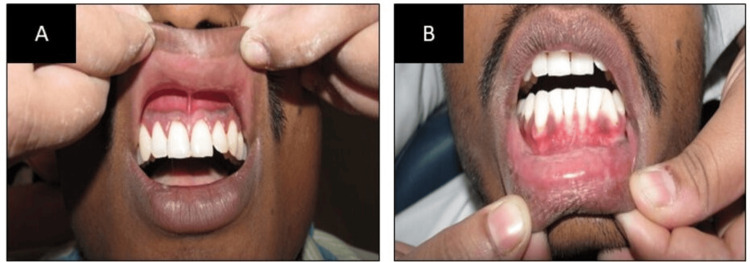
(A) Post-treatment photo of the upper anterior region of the jaw; (B) post-treatment photo of the lower anterior region of the jaw

Based on clinical and laboratory results, the patient was diagnosed with ANUG, and in the emergency, phase swabbing of the gingiva with H_2_O_2_ (hydrogen peroxide) swabs was done. The patient was prescribed metronidazole 400 mg twice a day for seven days and combiflam twice a day for five days as systemic antibiotics. Additionally, a 3% H_2_O_2_ rinse diluted 1:1 in warm water was recommended every two hours for three days. He was advised to discontinue the use of a mechanical toothbrush to prevent bleeding and pain. After three days, supragingival scaling was completed, and the bleeding and pain had stopped. The patient received counseling to stop betel squid, proper stress management, and instructions to resume oral hygiene. On the seventh day, the patient was advised to discontinue using the H_2_O_2_ rinse following another supragingival scaling procedure. It was noted that the gingival appearance was nearly restored (Figure 2). During the follow-up after one month, the patient showed signs of good oral hygiene.

## Discussion

Generally, ANUG is observed in young populations between 20 and 24 years old. It predominantly affects the anterior maxillary and mandibular gingiva. This infection is rare, although in developing countries, a prevalence of up to 25% is seen [5]. According to Listgarten et al. (1965 and 1967), using transmission electron microscopy, spirochetes were discovered in the unaffected connective tissue beneath necrotic gingival lesions in patients with ANUG. These spirochetes have been seen as far in advance of necrotic lesions as 400 μm from the basement membrane of the crevicular epithelium [6-9]. Glick et al. (1994) reported a correlation between ANUG and CD4+ T-lymphocyte counts below 200 cells, suggesting that doctors can make assumptions about the presence of ANUG based on CD4+ T-lymphocyte levels and vice versa [10,11].

The proposed protocol for the management of ANUG patients is different for different patients. The protocol for pediatric patients is mechanical debridement, followed by metronidazole 30 mg/kg for seven days, along with chlorohexidine mouthwash twice daily and professional cleaning with sonic instrumentation under conscious sedation. Post-treatment evaluation will be conducted through recall visits every three months, with follow-up for one year. For the adult population, the treatment is slightly modified. It also starts with gentle debridement and antibiotic drugs such as amoxicillin-clavulanate, clindamycin, or metronidazole combined with a macrolide. For scaling and root planning, it is recommended to use warm normal saline or a 3% peroxide solution as a mouth rinse. When managing HIV patients, the treatment generally starts with mechanical debridement and povidine iodine irrigation. The antibiotics prescribed are amoxicillin-clavulanate potassium 500 mg thrice a day for five days and metronidazole 500 mg thrice daily for five days. Furthermore, concurrent administration of antifungal medication, such as nystatin or clotrimazole, along with using chlorhexidine mouthwash twice daily and undergoing thorough scaling and root planning. Recall visits are scheduled every three and six months [5].

Additionally, it was found that young individuals with one or more predisposing factors, such as poor oral hygiene, physical or emotional stress, smoking, and weakened immunity to infection, are the primary victims of these diseases. Children who are undernourished exhibit more severe manifestations of the disease [1]. Stress has been identified as an important contributing factor to the development and aggravation of ANUG. ANUG lesions have the potential to develop into fatalistic outcomes or possibly cancrum oris (noma), a condition that is life-threatening if left untreated [4,12-14]. Oral lesions that may be mistaken for an ANUG include benign mucous membrane pemphigoid, human immunodeficiency virus, desquamative gingivitis, primary herpetic gingivostomatitis, and mouth ulcers linked to leukemia [1,15-18].

## Conclusions

The palliative care of the disease depends upon maintaining proper oral hygiene, quitting harmful habits, and counseling the patient mentally and physically. After a careful examination of the case, a treatment plan was planned. Immediate intervention was done according to the treatment plan, and antibiotic therapy was started immediately. Satisfactory results were achieved without any recurrence of the disease. This case report concludes that effective management of ANUG involves not only treating immediate symptoms and infections through professional dental treatments and improved oral hygiene but also improving patient physical health and underlying stress. By combining and addressing both the physical and psychological dimensions of the disease, long-term outcomes can be significantly improved, thus further reducing the reoccurrence and severity of ANUG.
